# Non-linear and context-dependent association of maternal BMI with cumulative live birth in Chinese women undergoing intrauterine insemination: a retrospective study of 3788 cycles

**DOI:** 10.3389/fendo.2026.1841344

**Published:** 2026-06-03

**Authors:** Huan-Qun Zhou, Jian-Fang Zhu, Ai-Ai Wang, Qi-Long Yuan, Ai-Hua Wu, Shan Lu, Wen Zhou

**Affiliations:** 1 Department of Reproductive Medicine, The Second Affiliated Hospital of Guangzhou University of Chinese Medicine, Guangzhou, China; 2 The Second Clinical College of Guangzhou University of Chinese Medicine, Guangzhou, China; 3 You Zhaoling National Studio for the Inheritance of Famous Traditional Chinese Medicine, The Second Affiliated Hospital of Guangzhou University of Chinese Medicine, Guangzhou, China; 4 Center for Reproductive Medicine, The 10th Affiliated Hospital of Southern Medical University (Dongguan People’s Hospital), Dongguan, China; 5 Guangdong Provincial Key Laboratory of Clinical Research on Traditional Chinese Medicine Syndrome, Guangzhou, China

**Keywords:** body mass index, cumulative live birth, interaction analysis, intrauterine insemination, non-linear association

## Abstract

**Background:**

Evidence regarding maternal body mass index (BMI) and intrauterine insemination (IUI) outcomes remains controversial. This study aimed to evaluate such independent associations, focusing on potential non-linear patterns, clinical thresholds, and population-specific heterogeneities.

**Methods:**

Data from 1951 couples (3788 cycles) were retrospectively analyzed. Multivariable generalized linear models (GLM), generalized estimating equations (GEE), and Cox proportional hazards models assessed first-cycle, per-cycle, and cumulative success, respectively; generalized additive model (GAM) and two-piecewise linear regression characterized non-linear patterns and thresholds.

**Results:**

While maternal BMI showed no significant independent association with clinical pregnancy or live birth in the first cycle (all *P* > 0.05), per-cycle analysis of 3788 cycles revealed a modest positive correlation (pregnancy: aOR 1.04, *P* = 0.004; live birth: aOR 1.03, *P* = 0.030). Notably, cumulative success followed a non-linear pattern (*P_LRT_* = 0.027), with live birth probability increasing until a BMI of approximately 21.2 kg/m² (aHR: 1.12, *P* = 0.007) but plateauing thereafter, potentially linked in part to an exploratory observation of higher spontaneous abortion rates in the obesity group (29.03%, *P* = 0.087). Subgroup analyses suggested potential heterogeneities in these associations across basal FSH levels and treatment protocols used in the first cycle (*P_interaction_* = 0.030 and 0.009, respectively). Specifically, for the FSH < 8mIU/mL group, a non-linear association was suggested (*P_LRT_* = 0.020), with success increasing up to approximately 21.2 kg/m² (aHR: 1.12, *P* = 0.011) and plateauing thereafter. Similarly, for patients whose first cycle used a gonadotropin protocol, a potential reversal was observed beyond approximately 21.0 kg/m² (*P_LRT_* = 0.020), where the trend shifted to a significant decline (aHR: 0.81, *P* = 0.023).

**Conclusions:**

Maternal BMI appears to exhibit a non-linear, context-dependent association with cumulative IUI success, underscoring the potential need for individualized preconception management.

## Introduction

Infertility currently affects approximately 15–20% of reproductive-aged couples globally, with IUI remaining a cornerstone first-line intervention due to its cost-effectiveness and relatively non-invasive nature (1–3). Among the clinical parameters influencing IUI success, female BMI is a key modifiable factor that essential for optimizing preconception counseling and treatment strategies. However, reports on the association between BMI and IUI outcomes remain inconsistent: while some evidence suggests that either elevated or low BMI is negatively associated with pregnancy outcomes (4–6), others have failed to observe any significant impact (7–9). These discrepancies may be attributed to the inherent heterogeneity across patient populations with diverse clinical characteristics (4, 5, 8, 9), as well as variations in outcome definitions ranging from per-cycle to cumulative results (6, 7). Furthermore, the conventional practice of categorizing BMI into broad intervals may mask more complex, non-linear relationships (4, 6, 9).

To address these uncertainties, we analyzed 1951 couples (3846 cycles) to evaluate BMI associations across first, per-cycle, and cumulative IUI attempts, with a focus on exploring potential non-linear relationships, identifying critical BMI thresholds, and uncovering heterogeneity across diverse clinical subgroups.

## Patients and methods

### Study population

**Figure 1:**
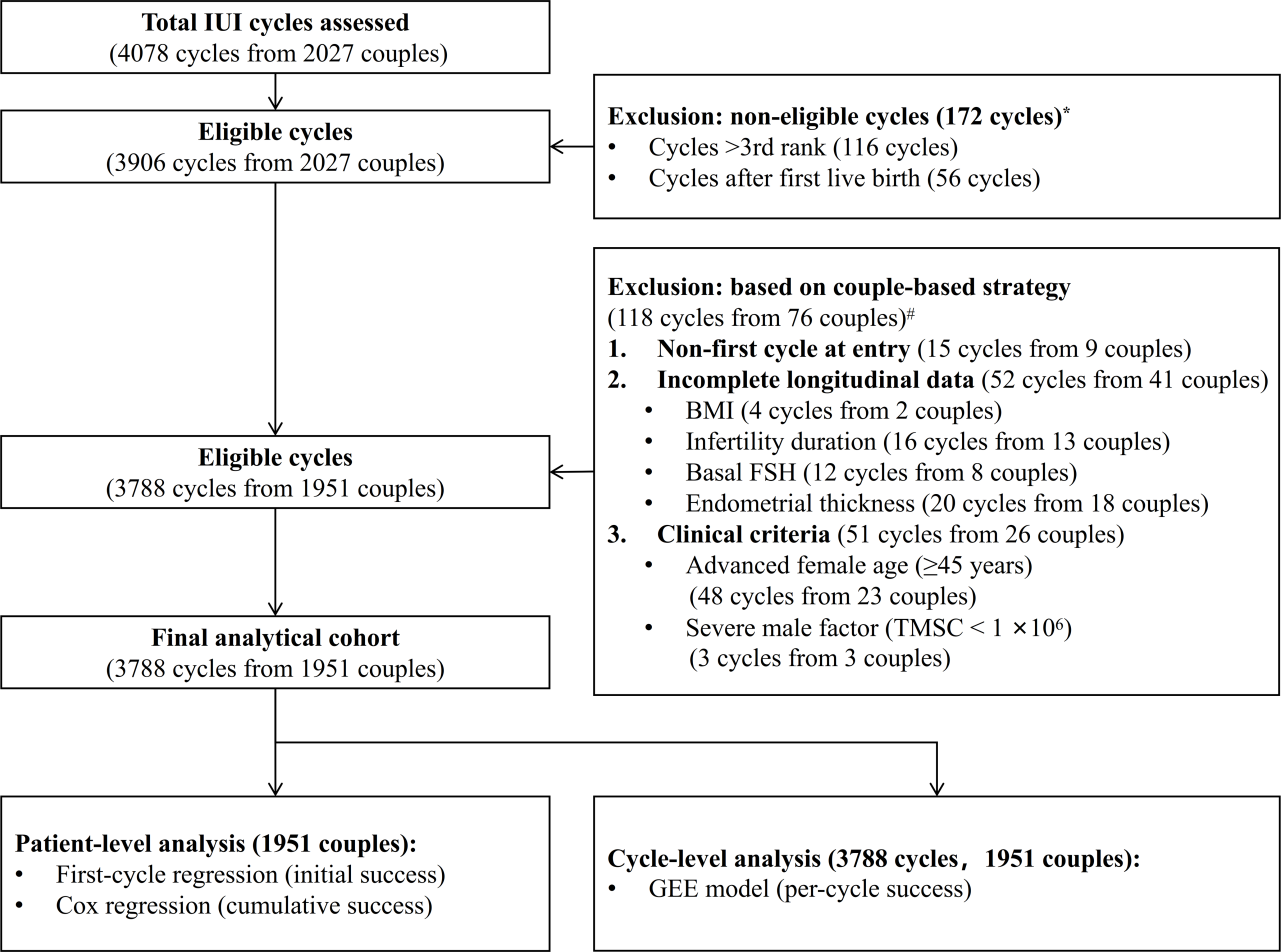
Flowchart of patient selection and study design. Stage 1 (Cycle-based)^*^: Only ineligible cycles were removed; all couples were retained based on their remaining eligible cycles. Stage 2 (Couple-based)^#^: Excluded couples meeting specific criteria, along with all their associated cycles. BMI, body mass index; FSH, follicle-stimulating hormone; TMSC, total motile sperm count after preparation; GEE, generalized estimating equation.

A total of 4078 consecutive IUI cycles (from 2027 couples), performed at the Reproductive Medicine Center of Guangdong Provincial Hospital of Chinese Medicine between January 2011 and December 2024, were initially identified. Following the study design, 172 non-target cycles were excluded (116 cycles beyond the third attempt and 56 cycles following a previous live birth). Notably, all 2027 couples were retained in the cohort at this stage, as they provided at least one eligible cycle within the study window. Subsequently, a couple-based exclusion strategy was applied: the entire longitudinal record was removed if a couple had a history of IUI prior to enrollment (15 cycles from 9 couples), missing key covariates—including BMI, infertility duration, basal FSH, or endometrial thickness—(52 cycles from 41 couples), or met clinical exclusion criteria such as maternal age ≥ 45 years or severe male factor (51 cycles from 26 couples). Consequently, a final cohort of 3788 cycles from 1951 couples was enrolled for analysis (**Figure 1**). This study was approved by the Institutional Review Board of Guangdong Provincial Hospital of Chinese Medicine (Ref No. ZE2025-469-01), with a waiver of informed consent for the retrospective analysis of de-identified data.

### IUI procedure

Ovarian stimulation was individualized using natural cycles or medicated protocols (clomiphene citrate, letrozole, or gonadotropins) initiated on menstrual cycle days 3–5. Follicular development was monitored via serial transvaginal ultrasound, integrated with urinary LH surge detection or serum hormone assays. Ovulation was triggered with human chorionic gonadotropin (hCG, 10,000 IU) or GnRH agonists when the lead follicle reached ≥18 mm (or two follicles ≥17 mm). To prevent multiple gestations, cycles with ≥ 3 dominant follicles were cancelled. IUI was performed 24–36 hours post-trigger. Semen samples were processed using density gradient centrifugation or the swim-up technique. A target post-wash total motile sperm count (TMSC) of ≥10×10^6^ was preferred; cycles with lower TMSC proceeded only after obtaining informed consent. Under ultrasound guidance, 0.3–0.5 mL of the sperm suspension was slowly inseminated into the uterine cavity. Luteal phase support, consisting of dydrogesterone or progesterone, was administered to patients with suspected luteal phase deficiency.

### Data collection

All clinical and laboratory data were retrieved from the center’s electronic IUI information system. These included patient demographics, infertility history, basal hormones, treatment protocols, endometrial thickness on the trigger day, post-wash TMSC, and pregnancy outcomes.

### Outcomes and definitions

The primary outcome was the live birth rate (LBR), defined as the delivery of at least one live-born infant at 28 weeks or more of gestation, which aligns with local clinical reporting practices. This was evaluated as: (1) First-cycle LBR: the live birth achieved in the initial IUI cycle; (2) All-cycle LBR: the live birth rate across all inseminated cycles, accounting for intra-couple correlation; and (3) Cumulative LBR (CLBR): the first live birth achieved within up to three consecutive cycles. Secondary outcomes included clinical pregnancy (defined as the visualization of at least one gestational sac, either intrauterine or extrauterine, via ultrasonography) and spontaneous abortion, which was defined as spontaneous pregnancy loss of an intrauterine pregnancy before 28 weeks of gestation. Other outcomes included ectopic pregnancy, preterm birth (delivery between 28 and <37 weeks), and neonatal outcomes (sex, birth weight, and congenital anomalies). The clinical pregnancy and live birth rates were calculated based on the total number of inseminated cycles. The ectopic pregnancy rate was calculated per clinical pregnancy, while the rates of spontaneous abortion and twin pregnancy were calculated specifically among intrauterine clinical pregnancies. Neonatal outcomes, including birth weight, sex, and congenital anomalies, were analyzed based on singleton live births to eliminate the confounding influence of plurality. To ensure data integrity, all patients were followed via telephone until delivery to record complete pregnancy and neonatal progress.

### Statistical analysis

Continuous variables (all non-normally distributed) were presented as medians (interquartile range, IQR) and analyzed via Kruskal-Wallis H tests, whereas categorical variables were expressed as frequencies (%) and assessed using chi-square or Fisher’s exact tests. The primary objective of this study was prognostic modeling to identify the independent predictive value of BMI for IUI outcomes within a real-world clinical context, rather than strictly formal causal inference. To assess clinical outcomes, multivariable generalized linear models (GLMs) were used for first-cycle analyses. For all-treatment-cycle data (n = 3788 cycles), generalized estimating equations (GEE) with a logit link function and an exchangeable correlation structure were employed to account for intra-couple correlation; in these models, time-varying covariates (e.g., treatment protocol, endometrial thickness, and TMSC) were updated for each specific cycle. For the three-cycle cumulative live birth rate (CLBR), multivariable Cox proportional hazards models (n = 1951 couples) were utilized to estimate independent associations; here, all covariates were defined based on the values from the first treatment cycle (baseline). Additionally, to validate the robustness of the Cox models against the discrete nature of IUI cycles, a discrete-time sensitivity analysis using pooled logistic regression was performed. Specifically, this model was based on a person-cycle dataset including only couples still at risk of their first live birth at the start of each cycle. The event indicator was defined as the occurrence of a live birth in that cycle, and the cycle number was entered as a categorical time variable. Furthermore, robust standard errors (clustered by couple ID) were used to account for repeated observations within couples. In these models, treatment-related covariates were updated per cycle. Covariates were retained based on clinical relevance, a >10% change-in-estimate of the BMI effect size, and the Events Per Variable (EPV) principle to ensure model stability and avoid overadjustment. To address the discrete nature of the IUI cycles (cycles 1, 2, 3) and the occurrence of tied event times, the Efron approximation was applied for parameter estimation. The proportional hazards assumption for Cox models was verified via Kaplan-Meier curves, which remained parallel and non-intersecting across cycles. Right-censoring was defined as failing to achieve a live birth after three cycles, treatment discontinuation, or loss to follow-up. While these cases were assumed to be non-informative for analysis, the potential for informative censoring—where discontinuation or loss to follow-up may relate to clinical prognosis—was explicitly acknowledged to ensure a cautious interpretation. Further analyses were focused on CLBR to explore complex associations. Specifically, generalized additive models (GAM) and two-piecewise linear regression identified non-linear patterns and threshold effects, respectively. Heterogeneity across clinical phenotypes was explored via interaction tests and subgroup analyses. For variables with significant interactions (*P_interaction_* < 0.05), stratified GAMs and threshold effect analyses were further performed to evaluate the stability of the detected non-linear patterns across different clinical strata. *Post-hoc* power analysis was conducted for primary and secondary outcomes (clinical pregnancy, live birth, and spontaneous abortion) to assess the statistical robustness of the comparison between normal weight and obesity groups (detailed in **Supplementary Table 1**). All statistical analyses were performed using EmpowerStats (version 6.0; X&Y Solutions, Inc., Boston, MA, USA) and R software (version 4.2.0; R Foundation for Statistical Computing, Vienna, Austria), with two-sided *P* < 0.05 considered significant.

## Results

### Baseline characteristics

**Table 1** summarizes the baseline characteristics of the 1951 participants during their first IUI cycles. The median BMI of the overall cohort was 21.21 kg/m². Significant differences across BMI categories were observed in age, infertility duration, antral follicle count (AFC), and basal FSH and LH (all *P* < 0.01). Notably, the prevalence of ovulatory disorder increased progressively with BMI, ranging from 35.74% in the underweight group to 66.27% in the obesity group (*P* < 0.001). Correspondingly, a higher frequency of ovarian stimulation was observed across these categories, rising from 57.38% to 78.31% (*P* < 0.001). However, no significant differences were observed among the four categories in terms of endometrial thickness (*P* = 0.980) or post-preparation TMSC (*P* = 0.487).

**Table 1:** Baseline characteristics of participants during their first IUI cycles according to BMI categories.

Characteristics	Overall (N=1951)	Underweight (n=305)	Normal weight (n=1259)	Overweight (n=304)	Obesity (n=83)	*P*-value
Female age, years	32 (30–36)	31 (29–35)	33 (30–36)	33 (30–36)	33 (31–36)	**<0.001**
Male age, years	34 (31–38)	33 (30–37)	34 (31–38)	35 (31–38)	35 (33–38)	**0.002**
BMI, kg/m²	21.21 (19.31–23.32)	17.75 (17.10–18.14)	21.03 (19.78–22.17)	25.16 (24.56–26.26)	30.01 (28.83–31.52)	**<0.001**
Infertility duration, years	2.50 (1.00–3.50)	2.50 (1.00–3.50)	2.500 (1.00–3.50)	2.50 (2.50–3.50)	3.50 (2.50–6.00)	**<0.001**
Basal FSH, mIU/mL	6.36 (5.43–7.54)	6.92 (5.90–8.13)	6.35 (5.45–7.52)	5.87 (5.16–6.97)	6.08 (5.15–6.94)	**<0.001**
Basal LH, mIU/mL	5.50 (3.84–7.59)	6.04 (4.33–8.39)	5.49 (3.85–7.41)	4.90 (3.45–7.39)	5.28 (3.60–7.42)	**<0.001**
AFC	18.00 (12.00–27.00)	18.00 (11.50–26.00)	18.00 (12.00–26.00)	22.00 (13.00–29.00)	22.00 (13.00–32.50)	**<0.001**
Endometrial thickness, mm	9.00 (7.700–11.00)	9.00 (8.00–11.00)	9.00 (7.90–11.00)	9.00 (7.20–11.00)	9.00 (7.00–11.25)	0.980
TMSC, ×10^6^	15.30 (10.00–24.25)	14.80 (10.20–24.20)	15.40 (10.10–24.10)	15.80 (9.80–26.00)	14.80 (8.45–22.30)	0.487
**Infertility type**						0.948
Primary	1376 (70.53%)	213 (69.84%)	893 (70.93%)	211 (69.41%)	59 (71.08%)	
Secondary	575 (29.47%)	92 (30.16%)	366 (29.07%)	93 (30.59%)	24 (28.92%)	
**Infertility cause**						**0.002**
Male	847 (43.41%)	132 (43.28%)	568 (45.12%)	127 (41.78%)	20 (24.10%)	
Female	371 (19.02%)	61 (20.00%)	226 (17.95%)	62 (20.40%)	22 (26.51%)	
Combined	589 (30.19%)	90 (29.51%)	360 (28.59%)	102 (33.55%)	37 (44.58%)	
Unexplained	144 (7.38%)	22 (7.21%)	105 (8.34%)	13 (4.28%)	4 (4.82%)	
**Specific etiology**						
Ovulatory dysfunction	771 (39.52%)	109 (35.74%)	453 (35.98%)	154 (50.66%)	55 (66.27%)	**<0.001**
Endometriosis	190 (9.74%)	38 (12.46%)	140 (11.12%)	8 (2.63%)	4 (4.82%)	**<0.001**
Prior miscarriage	230 (11.89%)	40 (13.20%)	137 (10.98%)	41 (13.62%)	12 (14.63%)	0.401
**Treatment protocol**						**<0.001**
Natural	728 (37.31%)	130 (42.63%)	499 (39.64%)	81 (26.64%)	18 (21.68%)	
Oral medications	597 (30.60%)	83 (27.21%)	378 (30.02%)	106 (34.87%)	30 (36.15%)	
Gonadotropins	626 (32.09%)	92 (30.16%)	382 (30.34%)	117 (38.49%)	35 (42.17%)	

Data are presented as median (IQR) or n (%). Missing data: n=7 for AFC and n=17 for prior miscarriage. BMI categories were defined as: underweight (<18.5 kg/m²), normal weight (18.5–23.9 kg/m²), overweight (24.0–27.9 kg/m²), and obesity (≥28.0 kg/m²). *P*-values were derived from the Kruskal-Wallis H test, chi-square test, or Fisher’s exact test, as appropriate. BMI, body mass index; FSH, follicle-stimulating hormone; LH, luteinizing hormone; AFC, antral follicle count; TMSC, total motile sperm count after preparation. Bold values indicate statistical significance (*P* < 0.05).

### Pregnancy and neonatal outcomes

Analysis of all 3788 cycles (**Table 2**) revealed that while the clinical pregnancy rate increased progressively with maternal BMI (11.68% to 20.81%, *P* = 0.008), the live birth rate showed a similar but non-significant trend (9.70% to 14.77%, *P* = 0.062). Notably, a non-significant trend toward higher spontaneous abortion was observed in the obesity group (29.03% *vs*. 10.61%–14.04% in other groups; *P* = 0.087). Regarding neonatal outcomes, exploratory analysis showed that gestational age showed a preliminary difference across categories (*P* = 0.020). For singletons, birth weight varied across groups (*P* = 0.002), with median values ranging between 3.02 kg and 3.40 kg. Other parameters, including neonatal sex, birth defects, and preterm birth rates, as well as the incidence of ectopic and twin pregnancies, were comparable across all BMI groups (all *P* > 0.05).

**Table 2:** Pregnancy and neonatal outcomes based on total IUI cycles according to BMI categories.

Outcomes	Overall	Underweight	Normal weight	Overweight	Obesity	*P*-value
**Cycle-based outcomes**	**(N=3788)**	**(n=608)**	**(n=2442)**	**(n=589)**	**(n=149)**	
Live birth rate	458 (12.09%)	59 (9.70%)	292 (11.96%)	85 (14.43%)	22 (14.77%)	0.062
Clinical pregnancy rate	564 (14.89%)	71 (11.68%)	360 (14.74%)	102 (17.32%)	31 (20.81%)	**0.008**
**Pregnancy-based outcomes**	**(n=564)**	**(n=71)**	**(n=360)**	**(n=102)**	**(n=31)**	
Ectopic pregnancy rate	28 (4.97%)	5 (7.04%)	18 (5.00%)	5 (4.90%)	0 (0.00%)	0.518
**Intrauterine pregnancy outcomes**	**(n=536)**	**(n=66)**	**(n=342)**	**(n=97)**	**(n=31)**	
Spontaneous abortion rate	76 (14.18%)	7 (10.61%)	48 (14.04%)	12 (12.37%)	9 (29.03%)	0.087
Twin pregnancy rate	37 (6.90%)	4 (6.06%)	27 (7.89%)	6 (6.19%)	0 (0.00%)	0.396
**Live birth-based outcomes**	**(n=458)**	**(n=59)**	**(n=292)**	**(n=85)**	**(n=22)**	
Plurality						0.728
Singleton	435 (94.98%)	56 (94.92%)	277 (94.86%)	80 (94.12%)	22 (100.00%)	
Twins	23 (5.02%)	3 (5.09%)	15 (5.14%)	5 (5.88%)	0 (0.00%)	
Gestational age (weeks)	39.50 (38.50-40.00)	39.00 (37.50-39.50)	39.50 (38.50-40.00)	39.50 (38.50-40.00)	38.50 (37.50-39.50)	**0.020**
Preterm birth rate	33 (7.21%)	5 (8.48%)	21 (7.19%)	5 (5.88%)	2 (9.09%)	0.923
**Singleton-based outcomes**	**(n=435)**	**(n=56)**	**(n=277)**	**(n=80)**	**(n=22)**	
Birth weight (kg)	3.19 (2.88-3.49)	3.02 (2.74-3.27)	3.19 (2.88-3.40)	3.40 (3.00-3.61)	3.14 (3.00-3.40)	**0.002**
Neonatal sex (Male)	216 (49.66%)	24 (42.86%)	139 (50.18%)	39 (48.75%)	14 (63.64%)	0.418
Birth defects	6 (1.38%)	0 (0.00%)	3 (1.08%)	3 (3.75%)	0 (0.00%)	0.206

Data are presented as median (IQR) or n (%). Missing data: n=1 for gestational age (total n=457). BMI categories were defined as: underweight (<18.5 kg/m²), normal weight (18.5–23.9 kg/m²), overweight (24.0–27.9 kg/m²), and obesity (≥28.0 kg/m²). *P*-values were derived from the Kruskal-Wallis H test, chi-square test, or Fisher’s exact test, as appropriate. Rates were calculated using the group-specific denominators indicated in the row headers. The difference between clinical pregnancies (n=564) and live births (n=458) is attributed to 76 spontaneous abortions, 28 ectopic pregnancies, and 2 stillbirths (at 28 and 29 weeks). Of the 37 twin pregnancies, 9 experienced spontaneous reduction to singletons, 4 resulted in complete abortion, and 1 resulted in stillbirth. BMI, body mass index. Bold values indicate statistical significance (*P* < 0.05).

### Association between BMI and pregnancy/neonatal outcomes

In first IUI cycles (**Table 3**), BMI was positively associated with clinical pregnancy (Crude OR: 1.05, *P* = 0.005) and live birth (Crude OR: 1.04, *P* = 0.048); however, these associations were not maintained in Model II (all *P* > 0.05). Relative to the normal weight group, no significant associations were observed between BMI categories and success rates (all *P* > 0.05). An observation of potential interest was the higher odds of spontaneous abortion in the obesity group (aOR: 3.55, 95% CI 1.12–11.29, 0.032). Given the small subgroup size and wide confidence interval, this finding is considered exploratory. Finally, exploratory analysis noted a correlation between BMI and singleton birth weight (β: 0.02 kg, *P* = 0.007), while other neonatal outcomes showed no significant associations (*P* > 0.05).

**Table 3:** Crude and adjusted associations between maternal BMI and outcomes in the first and total IUI cycles.

Outcomes	First cycle (N = 1951)			All cycles (N = 3788)		
	Crude	Model I	Model II	Crude	Model I	Model II
Pregnancy outcomes						
**Clinical pregnancy**	1.05 (1.02, 1.09) **0.005**	1.04 (1.00, 1.08) **0.034**	1.04 (1.00, 1.08) 0.070	1.06 (1.03, 1.09) **<0.001**	1.05 (1.03, 1.08) **<0.001**	1.04 (1.01, 1.07) **0.004**
Underweight	0.87 (0.60, 1.25) 0.446	0.87 (0.61, 1.26) 0.474	0.90 (0.62, 1.3) 0.56	0.77 (0.58, 1.00) 0.054	0.76 (0.58, 1.00) 0.053	0.79 (0.60, 1.04) 0.095
Overweight	1.18 (0.85, 1.65) 0.330	1.09 (0.78, 1.52) 0.629	1.06 (0.76, 1.49) 0.73	1.21 (0.95, 1.54) 0.120	1.15 (0.95, 1.47) 0.271	1.09 (0.85, 1.40) 0.483
Obesity	1.55 (0.90, 2.67) 0.115	1.38 (0.79, 2.38) 0.257	1.32 (0.75, 2.30) 0.334	1.52 (1.02, 2.26) **0.040**	1.41 (0.94, 2.11) 0.095	1.28 (0.85, 1.92) 0.236
**Spontaneous abortion**	1.07 (0.98, 1.17) 0.126	1.06 (0.97, 1.17) 0.194	1.07 (0.97, 1.18) 0.152	—	1.06 (0.95, 1.19) 0.316	—
Underweight	0.69 (0.23, 2.10) 0.509	0.67 (0.22, 2.07) 0.484	0.89 (0.28, 2.87) 0.852	—	—	—
Overweight	0.76 (0.29, 1.94) 0.561	0.66 (0.25, 1.75) 0.408	0.81 (0.30, 2.19) 0.683	—	—	—
Obesity	3.09 (1.05, 9.06) **0.034**	2.98 (1.00, 8.88) 0.050	3.55 (1.12, 11.29) **0.032**	—	—	—
**Live birth**	1.04 (1.00, 1.08) **0.048**	1.03 (0.99, 1.07) 0.140	1.03 (0.99, 1.07) 0.202	1.05 (1.02, 1.08) **0.001**	1.05 (1.02, 1.08) **0.003**	1.03 (1.00, 1.07) **0.030**
Underweight	0.91 (0.61, 1.35) 0.628	0.90 (0.61, 1.35) 0.620	0.92 (0.62, 1.38) 0.700	0.79 (0.59, 1.07) 0.123	0.78 (0.58, 1.05) 0.100	0.81 (0.60, 1.09) 0.164
Overweight	1.26 (0.88, 1.80) 0.213	1.17 (0.81, 1.68) 0.398	1.15 (0.80, 1.65) 0.460	1.24 (0.96, 1.61) 0.105	1.18 (0.91, 1.54) 0.218	1.12 (0.86, 1.47) 0.397
Obesity	1.22 (0.65, 2.31) 0.536	1.10 (0.58, 2.08) 0.771	1.08 (0.57, 2.06) 0.817	1.27 (0.80, 2.03) 0.308	1.19 (0.74, 1.92) 0.463	1.09 (0.67, 1.76) 0.729
Neonatal outcomes (singletons)						
**Gestational age (weeks)**	0.01 (-0.05, 0.07) 0.728	0.02 (-0.04, 0.07) 0.583	0.02 (-0.04, 0.08) 0.543	-0.001 (-0.04, 0.04) 0.948	0.001 (-0.04, 0.04) 0.976	0.003 (-0.04, 0.04) 0.878
Underweight	-0.30 (-0.87, 0.26) 0.296	-0.33 (-0.89, 0.24) 0.263	-0.28 (-0.85, 0.29) 0.340	-0.33 (-0.76, 0.10) 0.133	-0.34 (-0.78, 0.09) 0.118	-0.32 (-0.76, 0.12) 0.150
Overweight	0.11 (-0.39, 0.61) 0.654	0.14 (-0.36, 0.64) 0.586	0.18 (-0.33, 0.69) 0.484	0.15 (-0.18, 0.48) 0.371	0.16 (-0.18, 0.49) 0.358	0.17 (-0.15, 0.50) 0.298
Obesity	-0.27 (-1.14, 0.60) 0.541	-0.23 (-1.10, 0.65) 0.614	-0.21 (-1.09, 0.67) 0.638	-0.61 (-1.17, -0.05) **0.034**	-0.59 (-1.16, -0.03) **0.039**	-0.55 (-1.09, -0.02) **0.041**
**Birth weight (kg)**	0.02 (0.00, 0.04) **0.015**	0.02 (0.01, 0.04) **0.004**	0.02 (0.01, 0.03) **0.007**	0.02 (0.00, 0.04) **0.010**	0.02 (0.01, 0.03) **0.002**	0.02 (0.01, 0.03) **0.005**
Underweight	-0.15 (-0.31, 0.01) 0.067	-0.09 (-0.23, 0.05) 0.187	-0.08 (-0.22, 0.06) 0.263	-0.15 (-0.28, -0.01) **0.030**	-0.09 (-0.21, 0.02) 0.107	-0.08 (-0.19, 0.03) 0.164
Overweight	0.16 (0.03, 0.30) **0.021**	0.14 (0.02, 0.26) **0.025**	0.14 (0.02, 0.25) **0.025**	0.16 (0.03, 0.29) **0.014**	0.14 (0.02, 0.25) **0.020**	0.14 (0.02, 0.25) **0.023**
Obesity	-0.02 (-0.27, 0.22) 0.848	0.09 (-0.12, 0.30) 0.412	0.08 (-0.13, 0.29) 0.455	-0.02 (-0.24, 0.19) 0.832	0.09 (-0.09, 0.27) 0.339	0.08 (-0.10, 0.26) 0.383
**Neonatal sex (male)**	0.98 (0.90, 1.05) 0.532	0.98 (0.91, 1.06) 0.642	0.97 (0.90, 1.05) 0.498	1.02 (0.96, 1.08) 0.500	1.03 (0.97, 1.09) 0.409	1.03 (0.97, 1.09) 0.369
Underweight	1.22 (0.56, 2.63) 0.619	1.19 (0.55, 2.58) 0.660	1.28 (0.59, 2.81) 0.535	0.74 (0.41, 1.32) 0.307	0.71 (0.40, 1.28) 0.254	0.73 (0.41, 1.32) 0.296
Overweight	1.04 (0.53, 2.04) 0.918	1.07 (0.54, 2.10) 0.856	1.05 (0.52, 2.09) 0.902	0.94 (0.57, 1.54) 0.800	0.95 (0.57, 1.56) 0.825	0.97 (0.59, 1.61) 0.918
Obesity	0.95 (0.29, 3.07) 0.926	1.00 (0.31, 3.25) 0.993	0.94 (0.29, 3.12) 0.924	1.73 (0.70, 4.24) 0.235	1.77 (0.72, 4.33) 0.210	1.81 (0.73, 4.47) 0.200

Statistical Analysis: Data are presented as OR (95% CI) or β (95% CI) and P-values. ORs were calculated for categorical variables (pregnancy outcomes and neonatal sex), and β coefficients were reported for continuous variables (gestational age and birth weight). “First cycle” used multivariable logistic or linear regression; “All cycles” analyses used GEE with an exchangeable correlation structure to account for inter-cycle correlations within the same participant (grouped by medical record number). “—” indicates that the OR/CI and *P*-values could not be reliably estimated due to numerical non-convergence of the GEE model, typically resulting from sparse outcomes in specific BMI categories. BMI categories: underweight (<18.5 kg/m²), normal weight (18.5–23.9 kg/m², reference), overweight (24.0–27.9 kg/m²), and obesity (≥28.0 kg/m²). Sample Sizes: (1) Total participants/cycles: n=1951/n=3788; (2) Spontaneous abortion: n=285/n=530 (derived from intrauterine pregnancies after excluding cases with missing prior miscarriage history: 4 missing cases for the first cycle and 6 for all cycles, respectively); (3) Neonatal outcomes (singleton live births): n=231/n=434 for gestational age and birth weight analysis (after excluding 1 case with missing data for gestational age), and n=232/n=435 for neonatal sex analysis. Covariates: Clinical pregnancy & Live birth: Adjusted for female age, TMSC, and treatment protocol in Model I; and further adjusted for infertility duration, infertility factor, basal FSH, endometrial thickness and cycle sequence in Model II. Spontaneous abortion: Adjusted for female age and history of spontaneous abortion in Model I; and further adjusted for endometrial thickness and treatment protocol in Model II. Gestational age: Adjusted for female age in Model I; and further adjusted for endometrial thickness and treatment protocol in Model II. Neonatal sex: Adjusted for female age in Model I; and further adjusted for TMSC and treatment protocol in Model II. Birth weight: Adjusted for female age and gestational age in Model I; and further adjusted for treatment protocol and neonatal sex in Model II. BMI, body mass index; OR, odds ratio; β, regression coefficient; CI, confidence interval; GEE, generalized estimating equation; TMSC, total motile sperm count after preparation. For the “All cycles” GEE analysis, maternal BMI, female age, and basal FSH were analyzed as baseline measurements (first cycle values), as these factors were considered stable over the short duration of treatment. In contrast, cycle-specific covariates (treatment protocol, endometrial thickness, and TMSC) were updated and matched to each corresponding cycle to reflect their dynamic impact on outcomes. Bold values indicate statistical significance (*P* < 0.05).

In the GEE analysis of total cycles (**Table 3**), BMI was positively associated with clinical pregnancy (aOR: 1.04, *P* = 0.004) and live birth (aOR: 1.03, *P* = 0.030). Categorically, no significant associations were observed between BMI categories and the odds of clinical pregnancy or live birth relative to the normal weight group (all *P* > 0.05). Regarding spontaneous abortion, the GEE models for specific BMI categories, including the obesity group, did not reach numerical convergence due to sparse outcomes; however, a higher frequency was noted in the obesity group. For neonatal outcomes, exploratory analysis noted that BMI was associated with singleton birth weight (β: 0.02 kg, *P* = 0.005), with a suggestive reduction in gestational age in the obesity group (β: -0.55 weeks, *P* = 0.041).

### Association between BMI and cumulative live birth

In the Cox proportional hazards analysis (**Table 4**), BMI as a continuous variable was associated with cumulative live birth in the crude model (HR: 1.05, *P* = 0.001) and Model I (aHR: 1.04, *P* = 0.008). Notably, this association persisted after full adjustment in Model II (aHR: 1.03, *P* = 0.046). Categorically, no specific BMI category showed a significant association with cumulative live birth relative to the normal weight group (all *P* > 0.05). To assess the robustness of these findings, a sensitivity analysis incorporating the treatment period (categorized into 2011–2015, 2016–2019, and 2020–2024) as a covariate showed that the association remained stable (aHR: 1.03, 95% CI: 1.00–1.06, *P* = 0.034; **Supplementary Table 2**). Furthermore, discrete-time survival analysis using pooled logistic regression yielded consistent results (**Supplementary Table 3**), where BMI maintained its association as a continuous variable (OR: 1.03, 95% CI: 1.00–1.07, *P* = 0.030), while BMI categories showed no significant associations (all *P* > 0.05).

**Table 4:** Cox proportional hazards regression analysis for the association between BMI and cumulative live birth (n=1951).

Exposure	Crude		Model I		Model II	
	HR (95% CI)	*P*-value	HR (95% CI)	*P*-value	HR (95% CI)	*P*-value
**BMI (Continuous)**	1.05 (1.02, 1.08)	**0.001**	1.04 (1.01, 1.07)	**0.008**	1.03 (1.00, 1.06)	**0.046**
BMI Categories						
Normal weight	1.0 (Ref)		1.0 (Ref)		1.0 (Ref)	
Underweight	0.80 (0.61, 1.06)	0.126	0.79 (0.59, 1.04)	0.095	0.82 (0.62, 1.09)	0.164
Overweight	1.24 (0.97, 1.58)	0.081	1.15 (0.90, 1.46)	0.270	1.10 (0.86, 1.41)	0.434
Obesity	1.25 (0.81, 1.94)	0.305	1.14 (0.74, 1.76)	0.566	1.07 (0.69, 1.67)	0.752
**P for trend**	**0.007**		**0.032**		0.114	

Statistical Analysis: Data are presented as HR (95% CI) and *P*-values. All models were performed using Cox proportional hazards regression (n=1951). The outcome variable was cumulative live birth, and the time-to-event variable was the number of cycles. BMI categories: underweight (<18.5 kg/m²), normal weight (18.5–23.9 kg/m², reference), overweight (24.0–27.9 kg/m²), and obesity (≥28.0 kg/m²). Covariates: Adjusted for female age, TMSC, and treatment protocol in Model I; and further adjusted for infertility duration, infertility factor, basal FSH, and endometrial thickness in Model II. All covariates were defined based on the values from the first treatment cycle. BMI, body mass index; HR, hazard ratio; CI, confidence interval; TMSC, total motile sperm count after preparation. Bold values indicate statistical significance (*P* < 0.05).

### Subgroup analysis

Subgroup analyses were performed to evaluate the consistency of the association between BMI and cumulative live birth (**Table 5**). The positive association remained stable across most subgroups, with no significant interactions observed for maternal age, duration and type of infertility, endometrial thickness, TMSC, or ovulatory disorder (all *P_interaction_* > 0.05). Potential interactions were noted for basal FSH (*P_interaction_* = 0.030) and the treatment protocols used in the first cycle (*P_interaction_* = 0.009). Specifically, the association between BMI and cumulative live birth appeared more evident in patients with higher basal FSH levels (≥ 8 mIU/mL; aHR: 1.13, *P* = 0.006) and those whose first cycle followed a natural-cycle protocol (aHR: 1.08, *P* = 0.006) or an oral medication protocol (aHR: 1.06, *P* = 0.015).

**Table 5:** Subgroup analysis of the association between BMI and cumulative live birth.

Subgroups	N	HR (95%CI)	*P*-value	*P* for interaction
**Total**	1951	1.03 (1.00, 1.06)	**0.046**	–
**Maternal Age (years)**				0.613
<35	1310	1.024 (0.99, 1.06)	0.160	
≥35	641	1.04 (0.99, 1.11)	0.132	
**Duration of Infertility (years)**				0.571
<3	1121	1.04 (1.00, 1.08)	0.064	
≥3	830	1.02 (0.98, 1.06)	0.360	
**Type of Infertility**				0.935
Primary	1376	1.03 (1.00, 1.06)	0.081	
Secondary	575	1.03 (0.98, 1.08)	0.309	
**Infertility Factors**				0.645
Male	847	1.05 (1.00, 1.10)	0.080	
Female	371	1.03 (0.97, 1.09)	0.354	
Combined	589	1.01 (0.97, 1.06)	0.549	
Unexplained	144	1.09 (0.96, 1.25)	0.189	
**Ovulatory disorder**				0.073
No	1180	1.06 (1.01, 1.10)	**0.018**	
Yes	771	1.00 (0.97, 1.04)	0.902	
**Basal FSH** (mIU/mL)				**0.030**
<8	1601	1.02 (0.99, 1.05)	0.207	
≥8	350	1.13 (1.04, 1.24)	**0.006**	
**Treatment protocols in the first cycle**				**0.009**
Natural	728	1.08 (1.02, 1.14)	**0.006**	
Oral Medication	597	1.06 (1.01, 1.11)	**0.015**	
Gonadotropins	626	0.98 (0.94, 1.02)	0.375	
**Endometrial Thickness (mm)**				0.992
<8	503	1.03 (0.98, 1.08)	0.285	
≥8	1448	1.03 (1.00, 1.06)	0.093	
**TMSC (×10^6^)**				0.596
<10	483	1.02 (0.96, 1.07)	0.569	
≥10	1468	1.03 (1.00, 1.07)	0.052	

Statistical analysis: Data are presented as HR (95% CI) and *P*-values. All models were performed using Cox proportional hazards regression (n=1951), and the time-to-event variable was the number of cycles. BMI was analyzed as a continuous variable. *P* for interaction was calculated to assess the consistency of BMI’s effect across subgroups. Covariates: Adjusted for female age, infertility duration, infertility factor, basal FSH, endometrial thickness, TMSC, and treatment protocol, except for the variable used for stratification in each subgroup. All covariates were defined based on the values from the first treatment cycle. BMI, body mass index; HR, hazard ratio; CI, confidence interval; FSH, follicle-stimulating hormone; TMSC, total motile sperm count after preparation. All subgroup analyses were exploratory, and P-values were not adjusted for multiple comparisons. Bold values indicate statistical significance (*P* < 0.05).

### Non-linear relationship and threshold effect analysis

**Figure 2:**
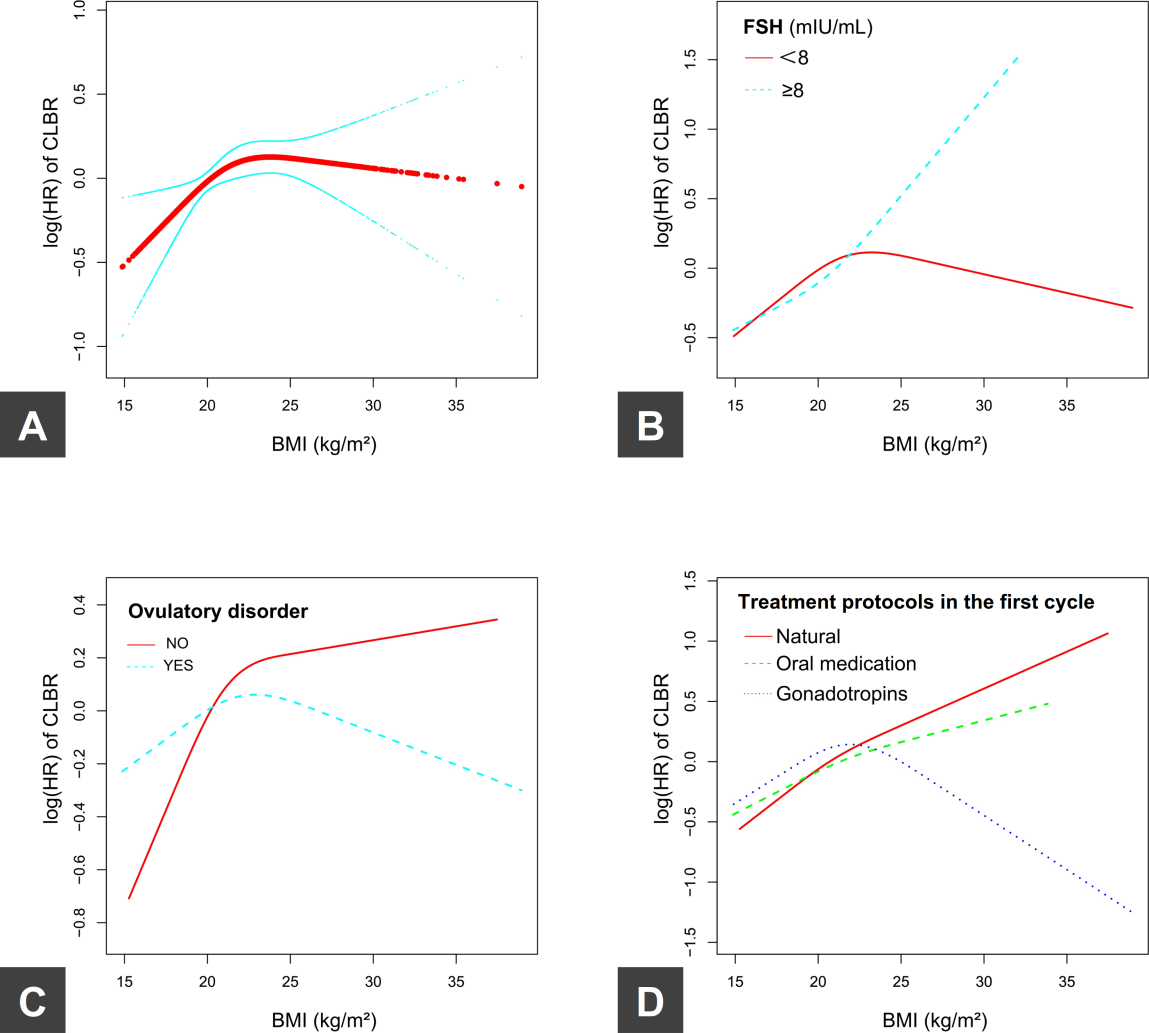
Non-linear association between BMI and cumulative live birth.Note: **(A)** Overall cohort; **(B)** Stratified by FSH; **(C)** Stratified by Ovulatory Disorder; **(D)** Stratified by Treatment Protocols. Statistical analysis: The solid red lines represent the smooth curve fits between BMI and the log-relative hazard of cumulative live birth based on GAM. The blue/cyan shaded areas or dashed lines represent the 95% CI. Covariates: Adjusted for female age, infertility duration, infertility factor, basal FSH, endometrial thickness, TMSC, and treatment protocol, except for the variable used for stratification in each subgroup. All covariates were defined based on the values from the first treatment cycle. GAM, generalized additive model; HR, hazard ratio; CI, confidence interval; BMI, body mass index; CLBR, cumulative live birth rate; TMSC, total motile sperm count after preparation; FSH, follicle-stimulating hormone.

GAM analysis revealed a non-linear association between BMI and the probability of cumulative live birth in the total population (**Figure 2**). A two-piecewise linear model identified a significant threshold effect (*P_LRT_* = 0.027) with an approximate inflection point at 21.2 kg/m² (**Table 6**). While numerical non-convergence in resampled datasets limited the estimation of a formal bootstrap confidence interval, the threshold’s existence was supported by the Likelihood Ratio Test. Below this approximate threshold of 21.2 kg/m², BMI was positively associated with cumulative live birth (aHR: 1.12, *P* = 0.007), whereas the association plateaued once BMI exceeded this value (aHR: 0.99, *P* = 0.774). Subgroup threshold analyses further characterized these divergent patterns. For patients without ovulatory disorders, those with basal FSH ≥ 8 mIU/mL, and those whose first cycle used a natural-cycle or oral medication protocol, BMI exhibited a consistent linear positive association throughout the observed range (all *P* < 0.05; *P_LRT_* > 0.05). In contrast, a robust threshold effect was identified in the basal FSH < 8 mIU/mL group, with an approximate inflection point of 21.2 kg/m² (*P_LRT_* = 0.020). Notably, among patients whose first cycle used a gonadotropin protocol (*P_LRT_* = 0.020), a suggestive positive trend existed below an approximate threshold of 21.0 kg/m^2^ (aHR: 1.14, *P* = 0.066), followed by a significant inverse association beyond this threshold (aHR: 0.81, *P* = 0.023). Given the exploratory nature of these subgroup analyses, these identified thresholds should be interpreted with caution.

**Table 6:** Threshold effect analysis of BMI on cumulative live birth in the total population and clinical subgroups.

Subgroups	N	Best fit model	Inflection point (K)	BMI range (kg/m^2^)	Adjusted HR (95% CI)	*P*-value	LRT *P*
**Total population**	1951	Piecewise	21.2	≤21.2	1.12 (1.03, 1.22)	**0.007**	**0.027**
**Basal FSH** (mIU/mL)				>21.2	0.99 (0.95, 1.04)	0.774	
<8	1601	Piecewise	21.2	≤21.2	1.12 (1.03, 1.23)	**0.011**	**0.020**
				>21.2	0.98 (0.94, 1.03)	0.387	
≥8	350	Linear	–	Overall	1.13 (1.03, 1.24)	**0.010**	0.383
Ovulatory Disorder							
No	1180	Linear	–	Overall	1.06 (1.01, 1.11)	**0.013**	0.053
Yes	771	Linear	–	Overall	1.00 (0.97, 1.04)	0.869	0.126
Treatment protocols in the first cycle							
Natural	728	Linear	–	Overall	1.08 (1.02, 1.15)	**0.008**	0.223
Oral Medication	597	Linear	–	Overall	1.05 (1.00, 1.11)	**0.037**	0.497
Gonadotropins	626	Piecewise	21.0	≤21.0	1.14 (0.99, 1.31)	0.066	**0.020**
				>21.0	0.81 (0.68, 0.97)	**0.023**	

Statistical analysis: The threshold effect of BMI on cumulative live birth was evaluated using a two-piecewise Cox proportional hazards model, with the optimal inflection point (K) identified by the maximum likelihood method. In this model, the number of IUI cycles was utilized as the time-to-event variable. LRT *P* represents the *P*-value for the log-likelihood ratio test comparing the piecewise model with the linear model; *P* < 0.05 indicates a significant non-linear association. Covariates: Adjusted for female age, infertility duration, infertility factor, basal FSH, endometrial thickness, TMSC, and treatment protocol. All covariates were defined based on the values from the first treatment cycle. HR, hazard ratio; CI, confidence interval; BMI, body mass index; LRT, likelihood ratio test; TMSC, total motile sperm count after preparation; FSH, follicle-stimulating hormone. Bold values indicate statistical significance (*P* < 0.05).

## Discussion

In this study of 1951 couples, we evaluated the association between maternal BMI and IUI outcomes across three distinct dimensions: first-cycle, per-cycle, and cumulative success. Our results suggest a potential non-linear threshold effect, where the cumulative probability of live birth rises initially but appears to plateau at an approximate BMI threshold of 21.2 kg/m². Notably, this association with live birth appeared to show some heterogeneity across subgroups defined by basal FSH and the treatment protocols used in the first cycle. Although interpreted cautiously due to sparse data in the obesity group, the observed rise in pregnancy loss may increasingly offset the fertility benefits of rising BMI. Additionally, a trend toward increased singleton birth weight and shorter gestational age was observed in relation to higher BMI. In summary, our study noted a potential plateau in the cumulative live birth once maternal BMI reached approximately 21.2 kg/m². Given the exploratory nature of these findings and the limited sample size in the obesity group, these observations should be interpreted with caution.

The threshold effect identified by the GAM potentially reflects a transition between reproductive restoration and metabolic impairment. Our data show that the clinical pregnancy and live birth rates were at their lowest levels in the underweight group (**Table 2**), and the cumulative probability of live birth exhibited a continuous ascending trend until reaching an approximate inflection point of 21.2 kg/m² (**Table 6**), which is consistent with previous reports regarding the negative impact of low BMI on IUI success (6). Mechanistically, we speculate that these observations could potentially be linked to previously reported pathways such as impaired GnRH pulsatility (10) and compromised endometrial receptivity (11, 12). Presumably, the initial ascending trend likely reflects the gradual alleviation of this physiological suppression alongside improved energy reserves. This hypothesis is supported by our baseline data (**Table 1**), which showed that although the underweight group had a younger median age, their basal FSH levels and the proportion of patients using natural-cycle protocols in the first cycle were both higher.

However, beyond the approximate 21.2 kg/m² threshold, the positive association between BMI and cumulative live birth appears to level off, suggesting a potential shift in the clinical benefit of weight gain. In our cohort, while higher BMI was linked to increased pregnancy rates, this trend was concurrently accompanied by a higher frequency of spontaneous abortion in the obesity group (29.03%, **Table 2**). It is noteworthy that the obesity group in our study had a relatively small sample size, which reflects both the lower prevalence of obesity among Chinese women of reproductive age and the clinical practice of recommending weight loss before initiating IUI cycles (13, 14). Nevertheless, certain obese individuals—either those with favorable baseline markers or those unable to achieve weight loss—proceed with treatment. Due to statistical imprecision and non-convergence of GEE models, the higher miscarriage risk in the obesity group represents a suggestive association rather than a robust independent finding. This potential increase in pregnancy loss likely contributes to the observed “ceiling effect” in cumulative live birth rates at higher BMI levels. From a biological perspective, existing literature suggests that obesity-induced chronic inflammation and lipotoxicity may compromise oocyte quality (15–17), providing a plausible biological framework for the observed upward trend in spontaneous abortion, although this association remains suggestive due to our study’s statistical constraints (18).

Regarding perinatal outcomes, we observed a positive correlation between maternal BMI and neonatal birth weight, alongside a trend toward decreased gestational age in the obesity group, which is consistent with previous studies (19–22). However, these findings should be interpreted conservatively as exploratory. Since the analysis was restricted to live births, potential selection bias cannot be ruled out. Furthermore, due to the small sample size in the obesity group, our study was not adequately powered to detect definitive perinatal effects. Despite these statistical constraints, our observations are biologically plausible and align with the hypothesis that maternal adiposity might create a hyperinsulinemic and pro-inflammatory intrauterine environment, which promotes fetal overgrowth while simultaneously increasing the risk of preterm birth potentially due to placental insufficiency or inflammatory triggers (23, 24).

Our findings are consistent with several previous studies but identify a more specific threshold. For instance, subgroup and threshold effect analyses revealed that the relationship between BMI and IUI outcomes exhibits heterogeneity, primarily driven by ovarian reserve and treatment protocols used in the first cycle. Notably, a significant interaction was observed between BMI and basal FSH levels (*P_interaction_* = 0.030). For women with preserved ovarian reserve (FSH < 8 mIU/mL), a piecewise linear model was superior (*P_LRT_* = 0.020), pinpointing an approximate inflection point at 21.2 kg/m², beyond which the benefit of weight gain plateaued (**Figure 2B**). In contrast, for those with FSH ≥ 8 mIU/mL, success rates showed a linear upward trend (aHR: 1.13, *P* = 0.010). While 10 mIU/mL is the standard threshold for diminished ovarian reserve, we adopted a 8 mIU/mL cutoff to ensure adequate statistical power, as patients with FSH > 10 mIU/mL were underrepresented in our IUI cohort. The observed linear trend in this subgroup might suggest that individuals with early-stage reserve depletion could be relatively more sensitive to energy insufficiency. One possible hypothesis is that higher energy reserves might provide a degree of compensatory support in this specific physiological context (25). Furthermore, the impact of BMI appeared to vary depending on the treatment protocols used in the first cycle (*P_interaction_* = 0.009); the positive correlation remained robust in natural and oral medication cycles but diminished in gonadotropin-stimulated cycles, which showed a unique piecewise pattern with a significant downward reversal beyond approximately 21.0 kg/m². Interestingly, a positive trend in isolated male factor infertility (aHR: 1.05, *P* = 0.080) further suggests that BMI may be a more sensitive determinant when other female reproductive pathologies are absent. Regarding ovulatory disorders, although the interaction did not reach statistical significance (*P_interaction_* = 0.073), we observed a potential trend where the positive association between BMI and IUI success possibly tended to be more apparent in women without ovulatory disorders (aHR:1.06, *P* = 0.018). This preliminary observation might suggest that the potent pharmacological effect of ovulation induction could potentially override the subtle physiological constraints of low body weight, thereby masking the intrinsic influence of BMI. Notably, treatment-related covariates as baseline variables may not fully account for their temporal variations across cycles, potentially leading to evaluation bias. However, a sensitivity analysis updating these factors per cycle yielded consistent results, suggesting that our findings are relatively robust to such fluctuations across the three cycles.

Despite its strengths, this study has several limitations. First, its retrospective nature precludes establishing definitive causality; despite adjusting for various confounders, residual confounding from unmeasured factors—such as sperm DNA fragmentation, lifestyle habits, and weight fluctuations or medications (e.g., metformin) used before the start of the cycle—remains possible. Second, the obese subgroup (BMI ≥ 28 kg/m²) was relatively small, reflecting the specific demographic profile of the Chinese IUI population and the clinical recommendation of weight loss prior to treatment. To quantify this limitation, a *post-hoc* power analysis was conducted (**Supplementary Table 1**), which indicated that the sample size may be insufficient to detect certain clinical differences. This limited sample size led to wide confidence intervals in certain analyses (e.g., spontaneous abortion), which reduces the statistical precision of findings at the highest BMI range. Third, BMI is an imperfect proxy that fails to distinguish fat from muscle mass or capture dynamic markers like HOMA-IR. Fourth, the inflection point of 21.2 kg/m² is an exploratory estimate. While the non-linear trend is statistically significant, the exact numerical value may be influenced by our cohort’s data distribution. Therefore, it should be viewed as a clinical reference rather than an absolute cutoff. Fifth, adjusting for cycle-specific factors such as treatment protocol and endometrial thickness might carry a risk of overadjustment, as they could potentially act as mediators, which may lead to a more conservative estimation of the total effect of BMI. Furthermore, given their exploratory nature and the lack of adjustment for multiple comparisons, these subgroup findings should be interpreted with caution as exploratory. Finally, as a single-center study on a Chinese cohort, the generalizability of our findings to other ethnicities with distinct metabolic thresholds requires further validation. The identification of an approximate BMI threshold of 21.2 kg/m² in this population suggests that ethnicity-specific cycle parameters may be a relevant area for future research. Such considerations could potentially assist reproductive endocrinologists in providing more tailored preconception counseling.

## Conclusion

In conclusion, our findings suggest a potential non-linear association between maternal BMI and the probability of cumulative live birth in IUI, with a suggestive inflection point at approximately 21.2 kg/m². Beyond this range, the biological benefits of weight gain appear to reach a plateau. Furthermore, this association pattern appears to be modulated by ovarian reserve and the treatment protocols used in the first cycle, reflecting a degree of heterogeneity. These results suggest the exploratory role of BMI as a context-dependent prognostic factor, which may help in providing more individualized preconception counseling for couples undergoing IUI. However, further validation in larger cohorts is warranted.

## Data Availability

The raw data supporting the conclusions of this article will be made available by the authors, without undue reservation.
