# Supplementary material for: Non-linear and context-dependent association of maternal BMI with cumulative live birth in Chinese women undergoing intrauterine insemination: a retrospective study of 3788 cycles
Source: Front Endocrinol (Lausanne). 2026 Jun 3;17:1841344. doi: 10.3389/fendo.2026.1841344 (PMC13271875; doi:10.3389/fendo.2026.1841344)
Supplement: Supplementary file 1 [file Table1.doc]

**Supplementary Table S1. Post-hoc power analysis of clinical outcomes between normal weight and obesity groups**

| **Outcome Measures** | **Normal weight** | **Obesity** | ***P*-value** | **Post-hoc Power (1-β)** |
| --- | --- | --- | --- | --- |
| **Cycle-based outcomes** | n=2442 | n=149 |  |  |
| Clinical pregnancy rate | 360 (14.74%) | 31 (20.81%) | 0.045 | 0.421 |
| Live birth rate | 292 (11.96%) | 22 (14.77%) | 0.308 | 0.135 |
| **Intrauterine pregnancy outcomes** | n=342 | n=31 |  |  |
| Spontaneous abortion rate | 48 (14.04%) | 9 (29.03%) | 0.026 | 0.413 |

**Note:** *P*-values were calculated using Pearson's chi-squared test for pairwise comparisons. Post-hoc power was determined based on observed rates and sample sizes with a two-sided alpha of 0.05.

**Supplementary Table S2. Sensitivity analysis of the association between BMI and cumulative live birth accounting for chronological shifts (2011–2024) (n=1951)**

| **Exposure** | **Primary Model (Model II)** | | **Sensitivity Model (Model III)** | |
| --- | --- | --- | --- | --- |
|  | **HR (95% CI)** | ***P*-value** | **HR (95% CI)** | ***P*-value** |
| **BMI (Continuous)** | 1.03 (1.00, 1.06) | 0.046 | 1.03 (1.00, 1.06) | 0.034 |
| **BMI Categories** |  |  |  |  |
| Normal weight | 1.0 (Ref) |  | 1.0 (Ref) |  |
| Underweight | 0.82 (0.62, 1.09) | 0.164 | 0.82 (0.62, 1.09) | 0.163 |
| Overweight | 1.10 (0.86, 1.41) | 0.434 | 1.11 (0.87, 1.42) | 0.402 |
| Obesity | 1.07 (0.69, 1.67) | 0.752 | 1.10 (0.71, 1.71) | 0.673 |
| ***P* for trend** | 0.114 | | 0.093 | |

**Note:** Statistical Analysis: Data are presented as HR (95% CI) and *P*-values. All models were performed using Cox proportional hazards regression (n=1951). The outcome variable was cumulative live birth, and the time-to-event variable was the number of cycles. BMI categories: underweight (<18.5 kg/m²), normal weight (18.5–23.9 kg/m², reference), overweight (24.0–27.9 kg/m²), and obesity (≥28.0 kg/m²). Covariates: Model II (Primary): Adjusted for female age, infertility duration, infertility factor, basal FSH, endometrial thickness, treatment protocol, and TMSC. Model III (Sensitivity): Further adjusted for treatment periods (2011–2015, 2016–2019, and 2020–2024) based on Model II to account for potential chronological shifts in clinical and laboratory practices. BMI, body mass index; HR, hazard ratio; CI, confidence interval; TMSC, total motile sperm count after preparation.

**Supplementary Table S3. Discrete-time sensitivity analysis based on 3788 IUI cycles from 1951 couples**

| **Exposure** | **Non-adjusted** | **Adjust I** | **Adjust II** |
| --- | --- | --- | --- |
| **BMI (Continuous)**  (3788 cycles from 1951 couples) | **OR (95%CI), *P*** | **OR (95%CI), *P*** | **OR (95%CI), *P*** |
|  | 1.05 (1.02, 1.08), 0.001 | 1.04 (1.01, 1.08), 0.003 | 1.03 (1.00, 1.07), 0.030 |
| **BMI Category** |  |  |  |
| Normal  (2442 cycles from 1259 couples) | 1 | 1 | 1 |
| Underweight  (608 cycles from 305 couples) | 0.79 (0.59, 1.07), 0.123 | 0.78 (0.58, 1.05), 0.101 | 0.81 (0.60, 1.09), 0.168 |
| Overweight  (589 cycles from 304 couples) | 1.24 (0.96, 1.61), 0.104 | 1.18 (0.91, 1.54), 0.220 | 1.12 (0.86, 1.47), 0.400 |
| Obesity  (149 cycles from 83 couples) | 1.27 (0.80, 2.03), 0.312 | 1.19 (0.74, 1.90), 0.476 | 1.09 (0.67, 1.76), 0.723 |

**Notes:** The statistical analysis was performed using pooled logistic regression based on a person-cycle dataset (n = 3788 cycles from 1951 couples) to account for the discrete nature of the time-to-event data. Robust standard errors (clustered by couple ID) were utilized to account for the potential correlation of repeated observations within the same couple. **Non-adjusted** model was adjusted for the treatment cycle number only. **Adjust I** model was adjusted for cycle number, female age, TMSC, and treatment protocol. **Adjust II** model was further adjusted for infertility duration, infertility factors, basal FSH, and endometrial thickness. BMI, body mass index; OR, odds ratio; CI, confidence interval; TMSC, total motile sperm count after preparation.
